# The gut and lung microbiome across the TB disease spectrum

**DOI:** 10.3389/fmicb.2025.1643900

**Published:** 2025-09-23

**Authors:** Rubeshan Perumal, Anou M. Somboro, Juhi Tulsi, Sinaye Ngcapu, Kogieleum Naidoo

**Affiliations:** ^1^MRC-CAPRISA HIV-TB Pathogenesis and Treatment Research Unit, Doris Duke Medical Research Institute, University of KwaZulu-Natal, Durban, South Africa; ^2^Department of Pulmonology and Critical Care, School of Clinical Medicine, University of KwaZulu-Natal, Durban, South Africa; ^3^Department of Medical Microbiology, School of Laboratory Medicine and Medical Sciences, University of KwaZulu-Natal, Durban, South Africa

**Keywords:** gut and lung microbiome, dysbiosis, inflammation, recurrent TB, relapse, reinfection

## Abstract

Tuberculosis (TB) remains a major global health challenge, affecting approximately 10 million people annually. Susceptibility to infection by *Mycobacterium tuberculosis*, progression to TB, response to antimycobacterial chemotherapy, and the propensity to develop post-infectious sequelae have all been linked to a complex interplay of host and pathogen factors. Studies have revealed that communities of microorganisms colonize the human respiratory and gastrointestinal tracts and regulate regional immunity, with consequent effects on TB acquisition, progression, and resolution. An in-depth understanding of the multifaceted determinants of host susceptibility to TB, including the cross-talk between the host immune system and gut and lung microbiomes, could provide new insights into TB pathogenesis, treatment response, sequelae, and recurrence dynamics. This review explores the role of the gut-lung microbiome axis across the spectrum of TB pathogenesis, including microbial changes during and beyond TB treatment, and assesses their potential effect on treatment outcomes and the risk of TB recurrence.

## Introduction

**Figure 1:**
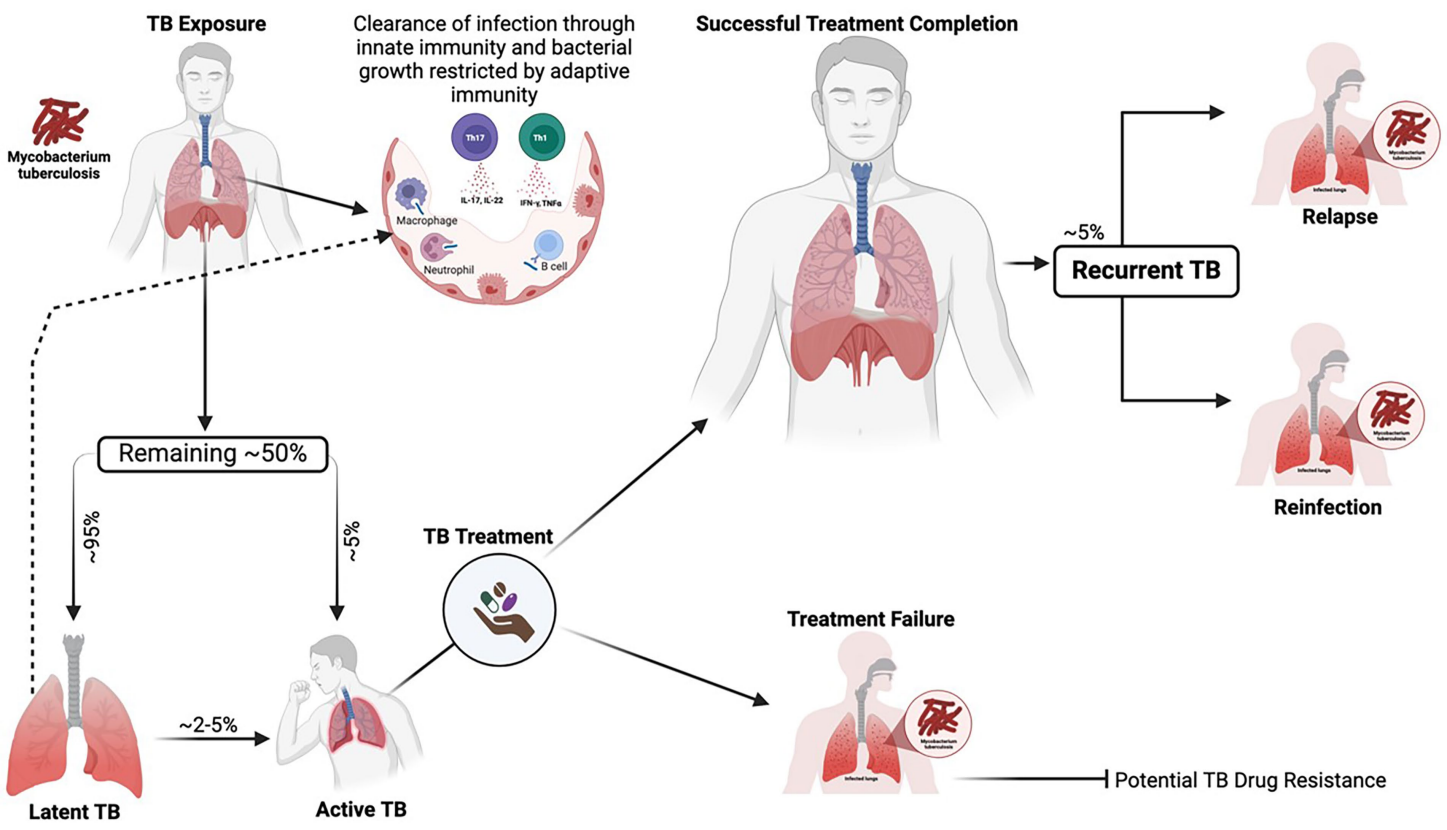
Dynamic spectrum of MTB infection depicting adequate containment, progression to active TB, treatment response, and TB recurrence. Upon exposure to MTB, the bacillus reaches the pulmonary alveoli, where many exposed individuals can clear the pathogen through innate immune responses, including the action of macrophages, natural killer cells, neutrophils, and cathelicidins. After the bacteria establish infection, adaptive immune mechanisms, such as cytokine production and the activation of CD4+ and CD8+ T cells, further restrict bacterial growth and help control the progression of the disease. The remaining individuals who fail to clear the infection may establish latent TB infection (~95%) or progress to active TB (~5%). Latently infected individuals may subsequently clear the infection or progress to active TB, most likely in the context of immunosuppression. Active TB can be successfully treated, but in some cases, there are therapeutic failures. Successfully treated individuals are at an increased risk of recurrent TB during their lifetime, whether through new infection (exogenous reinfection) or relapse of the initial disease episode (endogenous relapse). Generated with BioRender.com.

Tuberculosis (TB), caused by *Mycobacterium tuberculosis* (MTB), remains the leading cause of death from a single pathogen worldwide despite being a curable and preventable infectious disease (World Health Organization, 2024). In 2023, the number of new TB cases increased to over 10.8 million, further eroding the steady decline of approximately 2% per year until 2020. As the global TB burden remains unacceptably high, the current epidemiological trajectory is insufficient to meet the United Nations Sustainable Development Goals and the WHO End TB Strategy milestones for TB eradication (World Health Organization, 2024). TB control efforts are limited by unsatisfactorily high rates of treatment failure, which are associated with an increased risk of acquired drug resistance and mortality (World Health Organization, 2024). Additionally, after successful treatment completion, the risk of recurrent TB persists, either from reactivation of the initial MTB strain (relapse) or infection with a new strain (reinfection) (Colangeli et al., 2018; Romanowski et al., 2019; Jiang et al., 2022). Comorbidities such as HIV, diabetes, and malnutrition, as well as smoking and excessive alcohol use, are important risk factors for progression from latent infection to active TB disease and relapse (Bates et al., 2015; Duarte et al., 2018). TB susceptibility, progression, and relapse present significant public health challenges in resource-limited settings; however, the underlying determinants of these occurrences remain poorly understood (Figure 1).

Growing evidence indicates that dysbiosis of the gut and lung microbiota may influence susceptibility to *M. tuberculosis* infection, progression from latent tuberculosis infection (LTBI) to active TB, and response to anti-tuberculosis treatment (Dumas et al., 2018; Eribo et al., 2020; Wu et al., 2013). The gut microbiota may modulate: (i) interindividual variability in immune cell subsets or their functionality within the gut-lung axis, which can influence susceptibility to initial *M. tuberculosis* infection and the progression from latent to active disease; (ii) the absorption and therapeutic efficacy of antibiotics used in TB treatment; and (iii) the production of antimicrobial or immunomodulatory molecules capable of directly impacting *M. tuberculosis* growth and survival (Namasivayam et al., 2017; Comberiati et al., 2021). While evidence indicates a complex relationship between the gut and lung microbiome, host immune responses, and TB, the precise mechanisms underlying these interactions remain elusive. Additionally, key risk factors such as HIV infection, malnutrition, smoking, alcohol use, and diabetes are associated with significant structural and functional changes in the gut microbiota; however, the effects of these comorbidities and their clinical management on the gut-lung microbiome remain poorly understood. This review examines the gut-lung microbiome axis in TB pathogenesis, with a focus on microbial changes during and after TB treatment, and assesses their potential impact on treatment outcomes and the risk of TB recurrence. By examining these interactions, this review seeks to enhance our understanding of how microbial communities influence infection dynamics, treatment responses, and the likelihood of recurrence.

### Search strategy

Publications related to TB and the host microbiome were identified by searching PubMed, Google Scholar, and Web of Science using terms including but not limited to: (microbiota AND tuberculosis), (gut microbiome AND tuberculosis), (lung microbiome AND tuberculosis), (gut-lung axis AND microbiome AND tuberculosis), (tuberculosis treatment AND microbiome) and (recurrent tuberculosis AND microbiome). Further publications were identified from references cited in the relevant articles, reports, and conference proceedings. The search included all publications from inception until January 30, 2025, and was restricted to publications in or translated to English.

## Microbiome and TB susceptibility and progression

**Figure 2:**
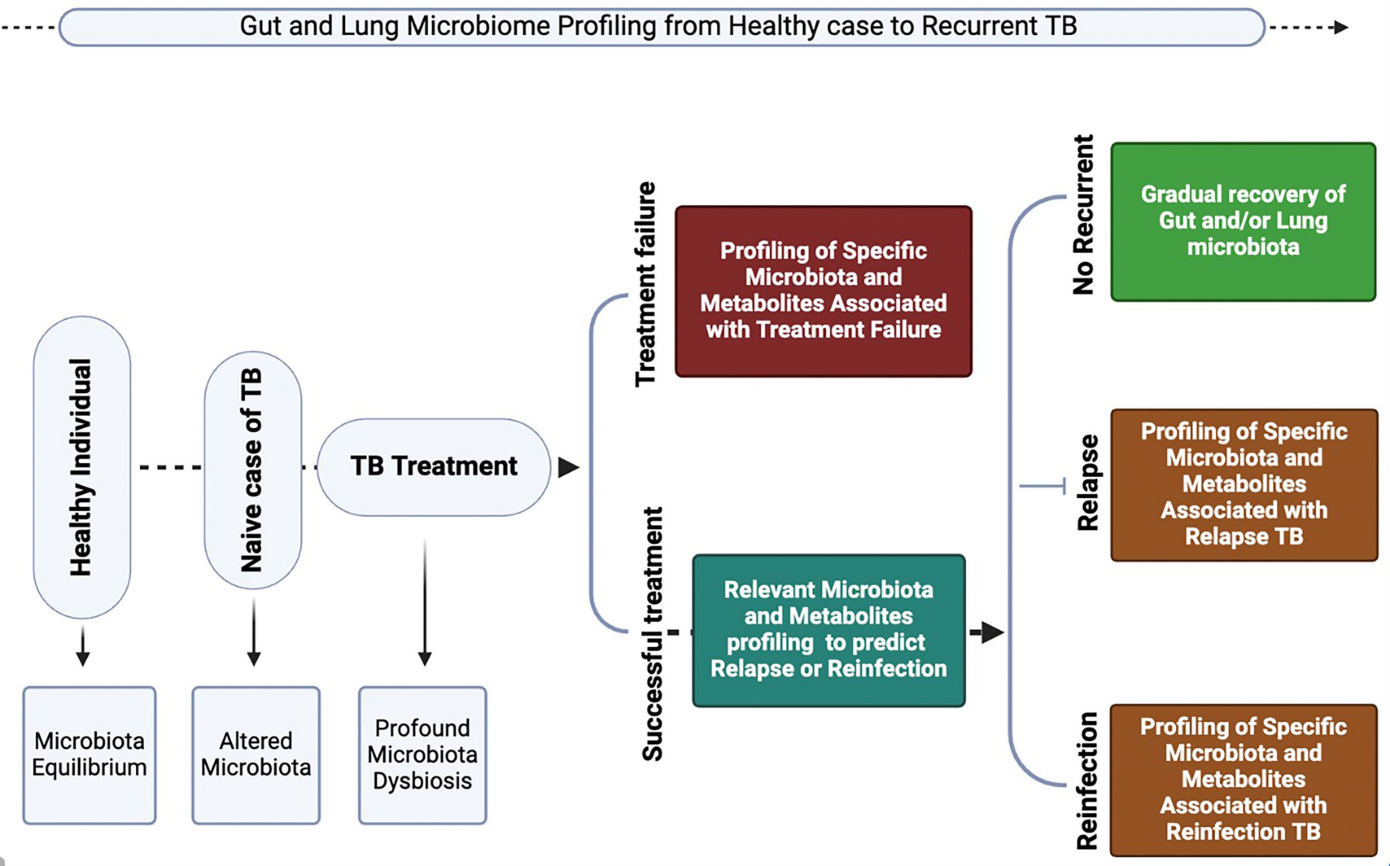
Crosstalk between the gut and respiratory microbiota during homeostasis and *Mycobacterium tuberculosis* (MTB)-induced dysbiosis. The schematic depicts the structural and immunological mechanisms underpinning the gut–lung axis. Left panels (homeostasis): In healthy conditions, epithelial integrity in both the intestine and lung is preserved by tight junctions and a protective mucus layer maintained by epithelial cell subsets, including secretory club cells (blue), goblet cells (green), ciliated cells (brown), basal cells (purple), and neuroendocrine cells (yellow). A balanced microbial community (Firmicutes, Bacteroidetes, and Proteobacteria) together with secretory IgA limits bacterial invasion. Antigen-presenting cells such as dendritic cells (DCs) and macrophages maintain immune equilibrium by stimulating naïve T cells (blue) and promoting a balance between proinflammatory effector T cells (purple/orange) and regulatory T cells (red). Cytokines released by these T cell subsets regulate Th1/Th17 activity, supporting effective lung immunity, microbial clearance, and epithelial barrier homeostasis. Right panels (MTB-induced dysbiosis): MTB infection perturbs epithelial integrity, resulting in leaky junctions and reduced mucus production. Dysbiosis is characterized by increased abundance of Proteobacteria and Bacteroidetes, reduced Firmicutes, and enhanced microbial translocation. Heightened antigen presentation amplifies proinflammatory T cell activation (purple/orange), while impaired cytokine regulation disrupts immune balance. This leads to defective lung immune responses, impaired MTB clearance, and enhanced microbial dissemination along the gut–lung axis, ultimately weakening host defense. Generated with BioRender.com.

The human microbiome, particularly the microbial communities in the lungs and gut, plays a pivotal role in immune regulation and host defense against infectious diseases (Ananya et al., 2021; Sencio et al., 2021; Schlechte et al., 2022; Du et al., 2023; Yang et al., 2024). In the context of TB, comprehensive studies on the microbiome composition in people with TB and MTB-infected animal models have highlighted that MTB may not solely drive TB pathogenesis. Instead, disruptions in microbial homeostasis and imbalances in the composition and abundance of microbiota in the lungs and gut can influence the host’s susceptibility to TB (Schlechte et al., 2022; Du et al., 2023; Nakhaee et al., 2018; Namasivayam et al., 2018; Naidoo et al., 2019; Zhou et al., 2021; Ma et al., 2022). Dysbiosis can impair the immune system’s ability to detect and respond effectively to MTB, thereby weakening host defense mechanisms. The lung microbiome plays a crucial role in shaping respiratory immune responses, whereas the gut microbiome has a broader impact on systemic immunity, which in turn modulates the host’s ability to resist TB infection (Ananya et al., 2021; Sencio et al., 2021; Schlechte et al., 2022; Du et al., 2023; Yang et al., 2024; Twigg 3rd et al., 2017; Balcells et al., 2019; Osei Sekyere et al., 2020). Therefore, understanding the complex interactions between these microbial communities and TB susceptibility may offer novel opportunities for targeted therapeutic interventions aimed at restoring the microbial balance and enhancing immune function to combat TB more effectively (Figure 2).

### Role of the lung microbiome in TB susceptibility and progression

Recent studies have demonstrated that the composition and diversity of lung microbial communities significantly increase an individual’s susceptibility to MTB; however, this relationship is complex and multifaceted. Advances in next-generation sequencing have revealed intricate interactions between the lung microbiome, MTB, and the alveolar immune system, highlighting the critical role these interactions play in determining the outcome of MTB infection (Adami and Cervantes, 2015). These interactions directly affect the lung immune system, which in turn can influence the development of resistance or susceptibility to MTB infection (Balcells et al., 2019; Adami and Cervantes, 2015). One key factor influencing susceptibility is dysbiosis, or a reduction in diversity within the lung microbiome, which creates a more favorable environment for MTB proliferation. This was evident in a study using a mouse model, in which disruption of the microbiota led to increased early colonization of the lungs by MTB during the first week of infection (Dumas et al., 2018). Interestingly, this disruption was also correlated with changes in the gut microbiota, suggesting a link between the gut and lung microbiomes in modulating susceptibility to MTB. Furthermore, microbiota depletion led to a reduction in mucosal-associated invariant T (MAIT) cells and CD8+ natural killer T (iNKT) cells in the lungs 1 week after infection, which are essential for immune defense and whose development is dependent on the host microbiota (Jabeen and Hinks, 2023). This reduction in MAIT cells, IL-17A production, and iNKT cells, which are crucial for immune defense against MTB, could contribute to the increased susceptibility to MTB (Im et al., 2008; Mihi and Good, 2019).

It has been hypothesized that lung dysbiosis depletes beneficial bacterial species that are important for immune function, thereby weakening the host’s ability to resist MTB colonization and proliferation. Studies have shown that MTB infection alters the microbial landscape of the lungs, leading to dysbiosis, disruption of oral airway boundaries, and shifts in lung microbial diversity. A meta-analysis of microbiota studies identified distinct bacterial species signatures associated with healthy controls (*Tumebacillus ginsengisoli*, *Propionibacterium acnes*, and *Haemophilus parahaemolyticus*) and TB patients (*Caulobacter henricii*, *Actinomyces graevenitzii*, and *Rothia mucilaginosa*), suggesting that lung microbiome composition may be associated with MTB infection outcomes (Hong et al., 2018). Additionally, specific oral-associated bacterial taxa, such as *SR1*, *Aggregatibacter*, *Leptotrichia*, *Prevotella*, and *Campylobacter*, are more abundant in MTB-infected individuals (Cadena et al., 2018). In individuals infected with MTB, the lung microbiome is dominated by MTB, whereas in MTB-negative individuals, the microbiome is more diverse and enriched with species such as *Streptococcus*, *Prevotella*, *Neisseria*, *Selenomonas*, and *Bifidobacterium* (Hu et al., 2020). These changes, combined with the depletion of *Helicobacter hepaticus*, *Firmicutes*, *Bacteroides*, and *Prevotella*, can impair immune regulation and reduce the ability to control MTB infection (Osei Sekyere et al., 2020; Yu et al., 2023). As a result, the host experiences a reduction in the expression of pathogen recognition receptors (e.g., macrophage-inducible C-type lectin, MINCLE) and MTB-killing cytokines (e.g., IFN-γ, TNF-α, and IL-17), leading to a weakened immune response and compromised phagocytic activity (Osei Sekyere et al., 2020). Furthermore, dysbiosis impairs autophagy, which is a crucial immune mechanism for clearing MTB and controlling inflammation. The reduction in microbiota-derived short-chain fatty acids (SCFAs), which are important for modulating immune cell function and limiting MTB proliferation, further exacerbates weakened immune defenses (Osei Sekyere et al., 2020). Similarly, another study demonstrated that TB patients exhibit a reduced diversity of alveolar microbiota compared with healthy controls, characterized by a decrease in *Streptococcus and Fusobacterium* species and an increase in mycobacterial abundance, which are potentially influenced by the inflammatory milieu, wherein MTB may release virulence factors such as ESAT-6 and CFP-10 to compete for nutrients, thereby suppressing the macrophage response (Mihi and Good, 2019; Vázquez-Pérez et al., 2020; Zhuo et al., 2023). Interestingly, commensal species such as *Lactobacillus plantarum* and microbiota metabolites, including indole propionic acid, have been shown to help control the progression of TB (Osei Sekyere et al., 2020; Negatu et al., 2018; Negatu et al., 2019). However, prior colonization by other pathogens, such as *Helicobacter hepaticus*, can disrupt the microbiota balance and reduce the body’s ability to resist MTB infection (Osei Sekyere et al., 2020; Majlessi et al., 2017).

Taken together, these findings suggest that a healthy and diverse lung microbiome may offer some protection against MTB, whereas dysbiosis creates an environment that is conducive to MTB colonization and proliferation. The relationship between the lung microbiome and MTB is highly complex and multifaceted, involving immune regulation, microbial interactions, and host factors. Further research is essential to fully understand these complex relationships and their potential implications for TB prevention and treatment (Twigg 3rd et al., 2017; Balcells et al., 2019; Osei Sekyere et al., 2020).

### Role of gut microbiota in TB susceptibility and progression

The gut microbiome, a highly complex and diverse community of microorganisms residing within the gastrointestinal tract, has garnered significant attention as a crucial factor that influences various aspects of human health and disease. Emerging evidence from recent studies have indicated that the gut microbiome plays a pivotal role in modulating susceptibility to MTB infection, and may influence both the progression and outcome of TB (Negi et al., 2019; Khan et al., 2016). The gut microbiota affects MTB susceptibility through multiple mechanisms, including via the gut-lung axis. The gut-lung axis is a bidirectional communication pathway that enables the gut microbiota to modulate immune responses in the lungs, potentially altering the host’s ability to resist MTB infection (Namasivayam et al., 2018).

Recent research involving both animal models and human subjects has provided evidence that MTB infection is associated with changes in the composition and metabolic function of the gut microbiota. However, these changes are not universally observed across all studies and may vary depending on the host’s genetic background, environmental factors, and the specific stage of the disease (Namasivayam et al., 2018; Hu et al., 2019; Li et al., 2019). Furthermore, the temporal relationship between MTB infection and alterations of the gut microbiome in human are incompletely understood.

Yang et al. investigated the role of the gut microbiota in MTB-infected mice by administering antibiotics to deplete the intestinal microbiota and found that the gut microbiota modulates host susceptibility to TB and pulmonary inflammatory responses through commensal gut bacteria-regulated long non-coding RNA (lncRNA-CGB) (Yang et al., 2022). Using a murine MTB infection model, lncRNA-CGB was shown to be down-regulated by antibiotic-induced dysbiosis of gut microbiota during MTB infection. To confirm the functional significance of lncRNA-CGB, a lncRNA-CGB genomic knock-out mouse strain was developed, and ultimately, it was confirmed that knock-out mice had higher infection rates and more severe disease than controls. The basis for this observed protection in lncRNA-CGB-replete mice is posited to be due to reduced IFN-γ expression and impaired anti-TB immunity associated with the down-regulation or knock-out of lncRNA-CGB. The study also revealed that *Bacteroides fragilis* (*B. fragilis*) plays a crucial role in enhancing lncRNA-CGB expression, inducing IFN-γ expression, and promoting protective immunity against MTB. Mechanistically, lncRNA-CGB interacts with enhancer of zeste homolog 2 (EZH2) to negatively regulate trimethylation of histone H3 lysine 27 (H3K27Me3) epigenetic programming, ultimately enhancing IFN-γ expression. In a separate study, Yang et al. demonstrated that gut dysbiosis can remotely increase micro ribonucleic acid 21 (miRNA-21) expression in the lung, resulting in a significant decrease in IFN-γ in mice treated with exogenous miRNA-21. Furthermore, dysbiosis-mediated miRNA-21 expression was found to enhance MTB infection and progression (Yang et al., 2020). In a C3HeB/FeJ mouse model, a high-fat diet was associated with dysbiosis, a low *Firmicutes/Bacteroides ratio*, *impaired immunological control of MTB following BCG vaccination*, and enhanced MTB proliferation (Arias et al., 2019). Broad-spectrum antibiotic-treated mice were also shown to experience severe dysbiosis, enhanced MTB proliferation, and more frequent dissemination of MTB to the liver and spleen (Khan et al., 2016). Antibiotic-induced dysbiosis was associated with suppressed Th1 cell release of IFN-γ and TNF-α, and augmented regulatory T-cell frequency (Khan et al., 2016). Interestingly, fecal transplantation in the antibiotic-treated mice led to improved MTB control and restoration of Th1 cell frequency and activity (Khan et al., 2016). *Helicobacter hepaticus* colonization in mice has been shown to lead to shifts in the gut microbiota, characterized by a predominance of *Bacteroides* and a decrease in *Firmicutes*, resulting in impaired immune control (innate immunity and accumulation of activated T cells) of MTB and greater lung tissue damage (Majlessi et al., 2017; Arnold et al., 2015). Furthermore, research in macaques has suggested that *Helicobacter pylori* infection reduces the risk of progression to active TB following exposure to MTB by increasing IFN-γ and Th1-like responses, implying a potential protective immunological effect.

Several studies have reported changes in the gut microbiota composition during MTB infection in mice, macaques, and humans, noting alterations in genera such as *Ruminococcaceae*, *Enterobacteriaceae*, *Erysipelotrichaceae*, and *Bifidobacterium* (Cadena et al., 2018; Winglee et al., 2014; Khaliq et al., 2021; Valdez-Palomares et al., 2021; Ding et al., 2022; Huang et al., 2023). MTB infection in mice has also been show to result in a reduction in the relative abundance of bacterial families, including *Clostridiales* (specifically *Lachnospiraceae* and *Ruminococcaceae*) and the *Bacteroidales* order, within 6 days of infection, with this shift persisting even in the absence of antibiotic treatment or detectable MTB DNA in fecal samples (Winglee et al., 2014).

While human data on the role of microbiota in the initial resistance to MTB infection remain limited, evidence suggests that commensal bacteria and their antimicrobial products may modulate resistance to various pathogens, including *Clostridium difficile*, *Salmonella enterica*, and vancomycin-resistant *Enterococcus* (Namasivayam et al., 2018). Notably, *Helicobacter pylori*, which infects millions of people globally, appears to have a protective effect against MTB infection. In keeping with this observation, individuals with LTBI who do not progress to active TB are more likely to be infected with *H. pylori*, supporting its potential role in conferring protection against TB (Perry et al., 2010). In pediatric populations, children with TB exhibit reduced gut microbiota diversity compared to healthy controls, with an upregulation of pro-inflammatory bacteria such as *Prevotella* and *Enterococcus* and downregulation of beneficial bacteria such as the *Bifidobacteriaceae* family (phylum *Actinobacteria*), *Ruminococcaceae* family, particularly SCFA-producing species *Faecalibacterium ruminococcaceae* and *Faecalibacterium prausnitzii* (order *Clostridiales*; phylum *Firmicutes*), and *Bacteroidaceae* family (phylum *Bacteroidetes*) (Li et al., 2019). Similarly, a study involving adult human subjects with active and latent tuberculosis infection (LTBI) revealed a slight decrease in the alpha diversity of the gut microbiota compared with healthy controls, particularly in the genus *Bacteroides* (phylum *Bacteroidetes*) (Hu et al., 2019). A clinical trial focused on gut microbiota diversity in patients with TB revealed a significantly lower diversity index in individuals with new or recurrent TB than in healthy controls. In these patients, the abundance of *Bacteroides* was reduced, whereas *Proteobacteria*, which includes many pathogenic species, was enriched. Interestingly, the genera *Prevotella* and *Lachnospira* were negatively correlated with peripheral CD4+ cell counts in the recurrent TB group, suggesting that specific gut microbes may influence the immune status and outcome of TB infection (Luo et al., 2017). These findings suggest that changes in the *Bacteroides* population may be a common feature of TB infection. As gut commensals, *Bacteroides* species contribute to host immunity by providing nutrients to other microbes and maintaining gut homeostasis through the release of outer membrane vesicles (OMVs).

Additionally, *Bacteroides* can modulate immune responses by secreting antimicrobial toxins and competing with pathogens (Hu et al., 2019; Zafar and Saier, 2021). Contrasting results were observed in a study conducted in Xinjiang, a multi-ethnic region, where the abundance of *Bacteroides* was found to be higher in people with TB than in individuals with LTBI or healthy controls (Wang et al., 2022). This discrepancy may reflect geographical differences, population genetics, or other environmental factors that influence microbial populations. Wang et al. reported alterations in fecal metabolites and microbial composition in TB patients, with genera such as *Bacteroides*, *Parabacteroides*, *Fusobacterium*, and *Lachnoclostridium* being enriched in people with TB, while *Blautia*, *Roseburia*, *Bifidobacterium*, and unidentified *Ruminococcaceae* were enriched in healthy individuals. Notably, this dysbiosis is associated with a reduction in SCFA production, particularly acetic, propionic, and butyric acids, which are known for their anti-inflammatory properties and role in regulating immune function (Qin et al., 2015; Wang et al., 2022).

In summary, these findings suggest that specific gut microbes may be closely associated with the host immune status, which in turn can influence the susceptibility, progression, and severity of TB. Shifts in the gut microbiota, such as changes in the abundance of specific microbial genera, including *Bacteroides*, *Prevotella*, and *Ruminococcaceae*, can modulate immune responses and potentially enhance or impair the host’s ability to control MTB infection. Futhermore, MTB infection may deleteriously alter the gut microbiome, promoting disease progression and dissemination. The influence of the gut microbiome on immune function highlights its potential role in determining whether TB remains latent or progresses to active disease, underscoring the importance of the microbiome in shaping TB outcomes.

## Microbiome and TB recurrence (relapse and reinfection)

There is a paucity of studies investigating gut and lung microbiome profiles in people who have successfully completed TB treatment, limiting our ability to assess the relationship between the end-of-treatment host microbiome and the subsequent risk of TB relapse or reinfection. The few published studies suffer from significant limitations, as many factors influencing the microbiome (e.g., age, nutrition, and environment) were not considered in the study design. Wu et al. profiled the lung microbiome associated with newly developed PTB, treatment failure, and recurrent TB (Wu et al., 2013). This study demonstrated that the genus *Pseudomonas*, a member of the phylum *Proteobacteria*, was more abundant in patients with treatment failure or recurrent TB than in new or recently cured TB patients and healthy individuals. *Pseudomonas* was also the only genus differentially abundant between cured TB patients and those who failed treatment. Furthermore, the relative abundances of *Prevotella*, *Bulleidia*, *Atopobium*, and *Treponema* were lower in patients with recurrent TB than in those with first episodes. The irregular repartition in abundance and frequency of these genera in recurrent TB and in patients failing treatment suggests that the disruption of normal bacterial flora in the respiratory tract could be a risk factor for treatment failure and TB recurrence (Wu et al., 2013). In a similar study, Luo et al. found distinct distributions of gut microbial communities among healthy individuals, newly diagnosed TB patients, and recurrent TB patients, characterized by an enrichment of the *Actinobacteria* and *Proteobacteria* phyla. The increased abundance of *Actinobacteria* and *Proteobacteria* suppresses the population of *Bacteroidetes* in patients with recurrent TB (Luo et al., 2017). This may indicate that in recurrent TB, there is a high risk of proliferation of pathogenic species favored by the phyla *Actinobacteria* and *Proteobacteria* at the expense of beneficial commensal microorganisms. However, these findings are yet to be confirmed by more robust and well-designed studies. Furthermore, Luo et al. showed that the relative abundance of *Prevotella* was positively correlated with the level of peripheral CD4 + cell counts in people newly diagnosed with TB, which was inversely correlated with recurrent TB. A similar trend was observed for the *Lachnospira* (Luo et al., 2017).

Despite the high rate of TB recurrence in endemic regions, studies investigating the microbiome as a biomarker for recurrent TB are rare. Conducting in-depth longitudinal studies examining the lung and gut microbiomes of newly diagnosed TB patients, treated patients, and those with recurrent disease would be informative. Such studies would help understand the key elements (e.g., structure, function, and abundance) of the microbiome, which may lead to therapeutic failure, TB recurrence, and acquisition of resistance. An earlier study highlighted the significance of evaluating lung microbiome parameters in TB infection, demonstrating that the *Pseudomonas/Mycobacterium* ratio was relatively elevated in recurrent TB cases compared to new TB cases. In contrast, the *Treponema/Mycobacterium* ratio was lower in recurrent TB cases, indicating that disruption of the complex healthy microbiome at the site of disease may be directly linked to TB recurrence (Wu et al., 2013). Interestingly, studies have identified distinct microbiome community structures in patients with multidrug-resistant TB (MDR-TB), and found the *Rothia*, *Delftia*, *Kingella*, *Chlamydophila*, and *Bordatella* genera to be potential biomarkers for the disease (Wiqoyah et al., 2021).

**Figure 3:**
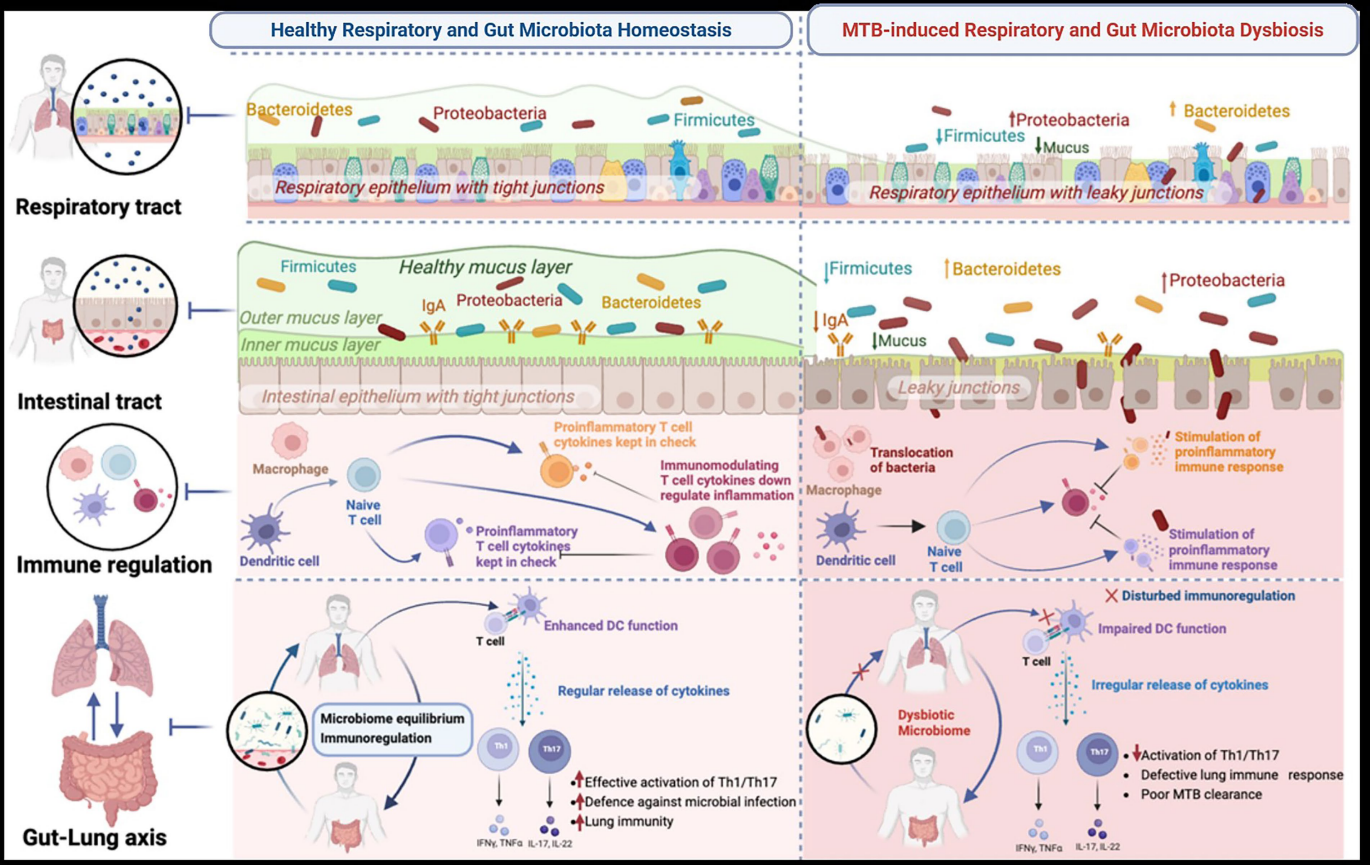
Illustration of the proposed applications of profiling gut and lung microbiota and metabolites, which could provide insights into microbiome biomarkers for predicting the onset of TB disease progression, treatment response, and recurrent TB (relapse or reinfection). Generated with BioRender.com.

The question remains: can the microbiome be actively managed to reduce progression to active TB, the risk of severe disease, and the risk of TB recurrence? First, profiling the gut and lung microbiome at different stages of TB will help illuminate the unique microbiota signatures of each TB state (Figure 3). Second, it will inform decisions on the reconstitution of these specific organs’ ecosystems, aiming to rectify not only the microbial structure but also the decline or overexpression of the immune cells involved in the defense against TB.

## Impact of comorbidities on the microbiome in TB

The influence of comorbidities on the TB microbiome has emerged as a significant area of research in recent years. Comorbidities, including HIV infection, diabetes, and malnutrition, are known to increase the risk of TB infection and influence disease progression. These conditions can also substantially alter the composition and function of the human microbiome, particularly in respiratory and gastrointestinal environments. The microbiome plays a critical role in modulating the immune response and overall health status of the host. Recent investigations have begun to elucidate the intricate relationships between comorbidities, microbiome, and TB pathogenesis.

### HIV-induced dysbiosis in TB

People living with human immunodeficiency virus/acquired immunodeficiency syndrome (PLWHA) are 26–31 times more likely to develop active TB than HIV-negative individuals, primarily due to the immunosuppressive effects of HIV on the host immune system (World Health Organization, 2012). Co-infection with HIV and MTB exacerbates the disease burden through immunological deterioration and HIV-induced dysbiosis, which increases the susceptibility to TB infection and accelerates disease progression. HIV-induced dysbiosis predominantly affects the gut microbiome, which plays a crucial role in immune regulation, with 70–80% of the immune cells residing in the gut-associated lymphoid tissue (Wiertsema et al., 2021). HIV disrupts the intestinal immune barrier, enabling microbial translocation and chronic systemic inflammation, both of which contribute to disease progression (Vujkovic-Cvijin et al., 2013). This disruption leads to gut dysbiosis, characterized by a reduction in microbial gene counts and an overabundance of gram-negative bacteria, acetogenic bacteria, and *Proteobacteria*, which can metabolize reactive oxygen and nitrogen species and potentially influence immune responses (Guillén et al., 2019). Disruptions in this axis may exacerbate TB progression and impair lung immunity, further contributing to the heightened susceptibility to TB observed in HIV-infected individuals.

In HIV-positive individuals with active TB, alterations in the lung microbiome may contribute to disease pathogenesis. Ueckermann et al. (2022) demonstrated that significant dysbiosis in a cohort of patients with HIV-associated TB, characterized by an increased abundance of *Burkholderia and Stenotrophomonas* in the lung microbiome of HIV-positive patients with active TB (Ueckermann et al., 2022). *Burkholderia and Stenotrophomonas* are commonly associated with underlying damage of the distal airways, especially in individuals with severe immunosuppression (Chawla et al., 2014; Huang et al., 2011; Regan and Bhatt, 2019). Similarly, in HIV-positive patients with pneumonia, a *Prevotellaceae*-dominated lower airway microbiome was found to independently predict mortality, with a distinct bacterial metabolome enriched in amino acid metabolites, monoacylglycerols, and inosine, along with elevated levels of pro-inflammatory cytokines such as IL-17A (Ueckermann et al., 2022). Further research into the impact of the microbiome on lung immunity revealed that, in PLWHA receiving antiretroviral therapy in a high-TB risk environment, the increased abundance of oral anaerobes, particularly *Prevotella*, in the lower airways correlated with higher serum levels of SCFAs (Segal et al., 2017). These elevated SCFA levels are associated with the induction of FoxP3-expressing Tregs in bronchoalveolar lavage lymphocytes following stimulation with a purified protein derivative (Segal et al., 2017; Zevin et al., 2016). These findings underscore the dual roles of gut and lung microbiomes in modulating immune responses to TB in HIV-infected individuals. The gut microbiome influences systemic immunity and the gut-lung axis, whereas alterations in the lung microbiome directly impact pulmonary immunity and host defense against TB. Targeting microbial dysbiosis in both the gut and lungs may represent a promising therapeutic strategy to improve immune responses and mitigate TB susceptibility in HIV-infected individuals. However, further research is required to elucidate the precise mechanisms by which gut and lung microbiome alterations contribute to TB pathogenesis and to identify effective interventions to address these microbial imbalances.

### Diabetes mellitus-induced dysbiosis in TB

The co-occurrence of TB and diabetes mellitus (DM) poses a significant global health challenge owing to the high disease burden of both conditions. One potential explanation for the heightened co-prevalence of these diseases is the alteration of immune and microbial responses to MTB infection driven by hyperglycemia in diabetic individuals. Studies have shown that TB patients with DM exhibit distinct changes in their lung microbial communities compared with non-diabetic patients (Hong et al., 2018; Hong et al., 2016). The combination of TB and DM is characterized by dysbiosis of the airway microbiota, which may play a role in the pathophysiological processes associated with TB disease (Hong et al., 2016). Interestingly, poor glycemic control in patients with diabetes and TB is associated with more severe TB symptoms and higher bacterial loads. Patients with HbA1C > 9% were significantly more likely to be smear-positive than non-diabetic patients, indicating a potential link between glycemic control and TB severity (Chiang et al., 2015). Hyperglycemia can lead to changes in lung microbiota and disrupt immune function by causing fluctuations in cytokine levels. This weakens the host’s ability to control MTB infection, increases susceptibility, and worsens the disease severity. Key cytokines associated with both hyperglycemia and lung dysbiosis, such as IFN-γ, IL-17, and TNF-α, play crucial roles in inflammatory pathways that influence the progression of TB (Mori et al., 2021). Thus, hyperglycemia may contribute to TB development by altering lung microbiota and impairing immune responses through cytokine imbalances. In patients with both DM and TB, elevated IL-6 levels are commonly observed, which may result from changes in the lung microbiome. The reduced presence of *Corynebacterium* in the lung microbiota is associated with higher IL-6 levels, highlighting its role in modulating lung immune responses (Liu et al., 2017). As IL-6 is a pro-inflammatory cytokine central to immune regulation (Uciechowski and Dempke, 2020), fluctuations in its levels are important for understanding the link between hyperglycemia and TB pathogenesis. In newly diagnosed DM-TB patients, IFN-γ, IL-2, IL-17, and TNF-α levels are lower, but tend to increase in patients with a history of DM-TB, likely due to the control of hyperglycemia following diabetes treatment (Li et al., 2020). Chronic hyperglycemia, rather than acute spikes, has a significant influence on lung inflammation through cytokines, such as IL-2, IL-17, and TNF-α (Ngo et al., 2021). IL-17 aids immune defense against MTB by recruiting neutrophils and promoting inflammation. In contrast, TNF-α and IL-2, which are essential for macrophage activation and adaptive immunity, are dysregulated by hyperglycemia in established DM-TB cases, potentially impairing immune tolerance, enhancing inflammatory responses, and compromising immune function, thereby exacerbating TB pathogenesis.

Emerging evidence has established a link between DM-related gut microbiota dysbiosis and altered host immune responses to MTB infection. However, the precise interactions between the gut microbiota composition and immune responses in the development of TB in patients with DM remain poorly understood. The relationship between DM and gut microbiota appears to be bidirectional; while diabetes can induce changes in gut microbiota composition, shifts in the microbiota may also affect glucose metabolism and energy homeostasis, potentially contributing to the pathogenesis of diabetes (Lv et al., 2018; Craciun et al., 2022). This interplay involves multiple mechanisms, including the production of metabolites such as SCFAs, branched-chain amino acids (BCAAs), and lipopolysaccharides (LPS) (Zhang et al., 2021; Hamamah et al., 2024). Comparative studies indicate that relative to healthy individuals, patients with DM exhibit a reduced abundance of genera such as *Bacteroides*, *Alistipes*, and *Blautia* (Zhu et al., 2020; Que et al., 2021; Liu et al., 2022), along with an increased presence of *Actinomyces* and *Prevotella* (Leite et al., 2017). Moreover, DM patients with LTBI demonstrate elevated levels of *Bacteroides*, *Alistipes*, and *Blautia* in contrast to those without LTBI, suggesting that LTBI status may be associated with microbiota dysbiosis in individuals with pre-existing DM. These microbiota alterations are implicated in modifications of metabolic, inflammatory, and immune functions, all of which play critical roles in the onset and progression of diabetes (Zhang et al., 2021). Taken together, these findings highlight the critical role of hyperglycemia in modulating immune responses in TB patients with DM, emphasizing the need for further research into the mechanisms by which metabolic conditions influence immune function to inform targeted therapeutic strategies.

## The gut and lung microbiome during TB treatment

The interaction between the microbiome and anti-TB drugs is complex and bidirectional, as the composition of the host microbiome can be influenced by drugs, and conversely, the microbiome can also influence an individual’s response to pharmacotherapy (Weersma et al., 2020). Thus, in addition to regulating the host immune defense against MTB, the host gut microbiome also affects the pharmacological parameters of anti-TB drugs, primarily by altering their pharmacokinetics and bioavailability through the production of enzymes that can transform the drug’s structure, thereby affecting its disposition and action (Meng et al., 2022). In addition to enzyme production, the microbiome can modify a drug’s pharmacokinetics and pharmacodynamics through changes in epithelial permeability, gut motility, intestinal drug transporters, and bile acids (Zhao et al., 2023).

Studies have shown links between microbiome alterations and the pathogenicity of pulmonary TB disease, particularly with respect to the impact of microbiome alterations on treatment response (Khan et al., 2019; Negi et al., 2020; Namasivayam et al., 2023). Using an *in vivo* murine model, Negi et al. demonstrated that the gut microbiome is a crucial determinant of isoniazid’s efficacy. The disparity in the gut microbial profile, characterized by an abundance of *Enterococcus* and a reduced population of *Lactobacillus* and *Bifidobacterium*, is associated with reduced isoniazid-mediated MTB killing, more aggressive tissue pathology, suppression of innate immunity (reduced antigen presentation and lower levels of interferon-gamma and TNF alpha), and mutation of T-cell responses against MTB (Negi et al., 2020). More recently, Namasivayam et al. demonstrated that alterations in the gut microbiome, such as those triggered by broad-spectrum antibiotic treatment, lead to significantly decreased bioavailability of rifampicin and moxifloxacin (Namasivayam et al., 2023). This implies that some anti-TB drugs will not be fully available for their intended biological targets and that the resultant low plasma concentration of these drugs could increase the risk of poor treatment outcomes (Perumal et al., 2020).

Conversely, some research findings have highlighted the disruption of gut microbiome composition, abundance, and function, partly attributable to the anti-TB drugs themselves (Namasivayam et al., 2017; Hu et al., 2019; Meng et al., 2022; Khan et al., 2019; Wipperman et al., 2017; Cao et al., 2021; Shi et al., 2021). Hu et al. found that anti-TB therapy induces rapid and significant alterations in the gut microbiota, with reduced alpha diversity and distinct community structure compositions before and after treatment (Hu et al., 2019). Members of the order *Clostridiales of the phylum Firmicutes [Firmicutes Ruminococcus* sp. *39BFAA, Ruminococcus gnavus, and Faecalibacterium (OTU 15)]*, which are vital for gut microbiome homeostasis and immune balance, mainly via the production of SCFAs, were significantly altered in individuals receiving anti-TB treatment. In contrast, many members of the phylum *Bacteroidetes* (e.g., *Bacteroides, Parabacteroides, Prevotella*), which produce polysaccharides that mediate mucosal tolerance through the upregulation of T-reg (Shen et al., 2012), were found to be over-represented during TB treatment (Hu et al., 2019; Cao et al., 2021). *Ruminococcus* and *Coprococcu*s are two genera that are significantly depleted in the gut microbiota during first-line TB treatment, and these two genera participate in the modulation of peripheral cytokine production (e.g., IL-1 and IFNγ) involved in the protective immune response against mycobacterial infection (Wipperman et al., 2017; Arpaia et al., 2013). Moreover, the reduction of other organisms, such as *Blautia, Lactobacillus, Bifidobacterium, Butyricicoccus, Acetivibrio, Alkaliphilus,* and *Peptococcus,* along with a simultaneous increase in Erysipeloclostridium, Verrucomicrobiaceae, and Fusobacterium, was observed in the gut microbiota of individuals receiving first-line anti-TB drugs (Namasivayam et al., 2017; Wipperman et al., 2017). *Lactobacillus* spp. influence the innate and adaptive immune responses by binding to pattern recognition receptors (PRRs) that detect bacterial and viral pathogens, *Bifidobacterium* spp. reduce Th17 cell activity, and *Prevotella* contributes to the Th17-mediated inflammatory response (Wells, 2011). The depleted immunologically significant commensal bacteria generally belong to the phylum *Firmicutes,* and other specific microbial genera and metabolites were also enriched or altered during treatment (Namasivayam et al., 2017; Meng et al., 2022; Khan et al., 2019; Wipperman et al., 2017; Cao et al., 2021; Shi et al., 2021). The phylum *Firmicutes* constitutes the most abundant microbiome in the gut (64%) and plays a crucial role in nutrition and metabolism (Piccioni et al., 2022). Therefore, the reduction of *Firmicutes* in the gut reveals intestinal homeostasis disruption, pathogen invasion, or pathological conditions that can affect treatment outcomes. Furthermore, TB therapy-derived gut microbiome dysbiosis is long-lasting and can persist for several months or even years in patients treated for MDR-TB (Wang et al., 2020). Treatment of drug-resistant TB requires the use of a diverse range of antibiotics for a prolonged period of time (~6–24 months), and has been shown to have a major impact on the community structure and richness of gut microbiota that persists for years (Wang et al., 2020). These changes to the microbiota are associated with increased *de novo* fatty acid synthesis and long-term changes to the systemic lipid profile (Wang et al., 2020; Shi et al., 2022).

Similar to its effect on the gut microbiome, TB treatment significantly affected alpha and beta diversity and disrupted the community structure of the lung microbiome. Several studies have reported distinct alterations in functional pathways after 1 month of anti-TB treatment (Kateete et al., 2021; Xiao et al., 2022; Zhang et al., 2022). For example, in a cohort study, Zhang et al. investigated the lung microbial composition and ecology, as well as the metabolic and functional pathways, to assess the impact of TB treatment (Zhang et al., 2022). *Firmicutes* was identified as the predominant taxa in the lungs of healthy and latently infected individuals. In contrast, a rapid and significant increase in the phylum *Bacteroidetes* and simultaneous depletion of the members of *Firmicutes* (e.g., *Subdoligranulum*, *Lactobacillus, Bulleidia* and *Allobaculum*) were observed in the lungs of TB patients who received a month of treatment. Compared to treatment-naïve patients, the treated group exhibited a significant reduction in functional pathways, including indole alkaloid biosynthesis, Wnt signaling pathway, endocytosis, and cytochrome P450 metabolism of antibiotics (Mori et al., 2021; Zhang et al., 2022). Kateete et al. also found that by the second month of treatment, first-line anti-TB drugs dramatically reduced the abundance of bacterial families, such as *Ruminococcaceae and Peptostreptococcaceae*, suggesting a discernible treatment-associated microbiota signal representing the potential for this to serve as a treatment monitoring biomarker (Kateete et al., 2021). Moreover, long-term exposure to anti-TB drugs in the lung microbiota accelerates the enrichment of antimicrobial resistance genes (ARGs) in patients with TB (Xiao et al., 2022). These changes suggest that disruption of the lung microbiome caused by anti-TB treatment may increase the likelihood of acquired drug resistance. Thus, understanding the mechanisms by which treatment-associated microbiome dysbiosis affects the predisposition to acquired drug resistance or disease recurrence is an important area for future investigation.

## Conclusion

The gut and lung microbiomes undergo structural and functional changes during TB infection, and these changes evolve in response to the disease state, treatment, and the onset of recurrence. Shifts in the microbiome affect metabolic, inflammatory, and immune pathways in people infected with TB, mediating the propensity for disease progression. Other factors, such as social conditions, diet, and genetic characteristics, also influence the integrity of the microbiome. Further consideration in future studies is required to obtain insights into the unique MTB-related gut and lung microbiome profiles at TB infection, during disease progression, in response to treatment, and prior to TB recurrence (Figure 3). To date, there is limited consensus regarding the structure and function of the microbiome in these dynamic disease states, and additional research is essential to comprehensively characterize the gut and lung microbiomes as biomarkers of distinct disease states and as targets for therapeutic intervention.

All authors contributed to the conceptualization, drafting, review, and finalization of the manuscript.
